# Heterogeneity of HER2 Expression in Circulating Tumor Cells of Patients with Breast Cancer Brain Metastases and Impact on Brain Disease Control

**DOI:** 10.3390/cancers14133101

**Published:** 2022-06-24

**Authors:** Douglas Guedes de Castro, Antônio Cássio Assis Pellizzon, Alexcia Camila Braun, Michael Jenwei Chen, Maria Letícia Gobo Silva, Ricardo Cesar Fogaroli, Guilherme Rocha Melo Gondim, Henderson Ramos, Elson Santos Neto, Carolina Humeres Abrahão, Liao Shin Yu, Emne Ali Abdallah, Vinicius Fernando Calsavara, Ludmilla Thomé Domingos Chinen

**Affiliations:** 1 Department of Radiation Oncology, A.C.Camargo Cancer Center, São Paulo 01509-010, Brazil; acapellizzon@accamargo.org.br (A.C.A.P.); michael.chen@accamargo.org.br (M.J.C.); leticia.silva@accamargo.org.br (M.L.G.S.); ricardo.fogaroli@accamargo.org.br (R.C.F.); guilherme.gondim@accamargo.org.br (G.R.M.G.); henderson.ramos@accamargo.org.br (H.R.); elson.neto@accamargo.org.br (E.S.N.); carolina.abrahao@accamargo.org.br (C.H.A.); 2 International Research Center, A.C.Camargo Cancer Center, São Paulo 01509-010, Brazil; abraun@acamargo.org.br (A.C.B.); emne22@gmail.com (E.A.A.); 3 Department of Imaging, A.C.Camargo Cancer Center, São Paulo 01509-010, Brazil; liao.yu@accamargo.org.br; 4 Samuel Oschin Comprehensive Cancer Institute, Cedars-Sinai Medical Center, Los Angeles, CA 90048, USA; viniciusfcalsavara@gmail.com; 5 Translational Medicine Laboratory, IBCC Oncology, São Paulo 03102-002, Brazil; ltdchinen@gmail.com

**Keywords:** circulating tumor cells, brain metastases, radiosurgery

## Abstract

**Simple Summary:**

Results from a previous study suggested that the number of circulating tumor cells (CTC) might have a role as a biomarker of early distant brain failure in patients with breast cancer brain metastases (BCBM). However, it remains largely underexplored whether heterogeneous HER2 expression in CTC may have a prognostic implication. We evaluated the status of HER2 expression in CTC before and after radiotherapy/radiosurgery for BCBM and observed that the presence of HER2 expression in any moment was associated with longer distant brain failure-free survival, irrespective of the primary immunophenotype of the breast tumor. This finding suggests that the status of HER2 expression in CTC has the potential to improve the treatment selection for patients with BCBM.

**Abstract:**

HER2 expression switching in circulating tumor cells (CTC) in breast cancer is dynamic and may have prognostic and predictive clinical implications. In this study, we evaluated the association between the expression of HER2 in the CTC of patients with breast cancer brain metastases (BCBM) and brain disease control. An exploratory analysis of a prospective assessment of CTC before (CTC1) and after (CTC2) stereotactic radiotherapy/radiosurgery (SRT) for BCBM in 39 women was performed. Distant brain failure-free survival (DBFFS), the primary endpoint, and overall survival (OS) were estimated. After a median follow-up of 16.6 months, there were 15 patients with distant brain failure and 16 deaths. The median DBFFS and OS were 15.3 and 19.5 months, respectively. The median DBFFS was 10 months in patients without HER2 expressed in CTC and was not reached in patients with HER2 in CTC (*p* = 0.012). The median OS was 17 months in patients without HER2 in CTC and was not reached in patients with HER2 in CTC (*p* = 0.104). On the multivariate analysis, DBFFS was superior in patients who were primary immunophenotype (PIP) HER2-positive (HR 0.128, 95% CI 0.025–0.534; *p* = 0.013). The expression of HER2 in CTC was associated with a longer DBFFS, and the switching of HER2 expression between the PIP and CTC may have an impact on prognosis and treatment selection for BCBM.

## 1. Introduction

The increasing detection of patients with breast cancer brain metastases (BCBM) is due to evolving diagnostic resources and systemic therapy, and has been accompanied by an improvement in overall survival (OS) [1,2]. In an effort to optimize therapeutic decisions, nomograms [3] and prognostic metrics [4,5] have been developed to predict distant brain failure (DBF).

Recently, a prospective study showed that the number of circulating tumor cells (CTC) may have a role as a biomarker of early DBF and a guide for salvage therapy after initial stereotactic radiotherapy/radiosurgery (SRT) [6]. In addition to the number, the phenotype of the CTC was evaluated in the same study, including the expression of human epidermal growth factor receptor 2 (HER2), along with other potential markers for the identification and characterization of CTC for breast cancer brain metastases [7,8].

The propensity to develop BCBM varies by the immunophenotype, occurring in approximately a third of patients with HER2-positive breast cancer [9]. Nevertheless, discordance in HER2 expression between the primary tumor and BCBM has been found in approximately 12% of patients [10]. Additionally, spontaneous interconversion between HER2-positive and negative expression in CTC and its potential prognostic and clinical implications has been reported [11], supporting the need for a reassessment of HER2 expression during the course of the breast cancer disease.

However, it remains largely underexplored whether heterogeneous HER2 expression in CTC may have prognostic and predictive clinical implications in patients with BCBM. In this study, we assessed the association between the status of HER2 expression in the CTC of patients with BCBM and its impact on brain disease control.

## 2. Materials and Methods

### 2.1. Patients and Study Design

Adult patients diagnosed with BCBM, with the primary immunophenotype (PIP) determined from a primary tumor in the breast, and candidates for SRT were invited to participate in this prospective study, which was approved by the institutional review board (no 2256/16). The exclusion criteria included pregnancy, whole-brain radiotherapy (WBRT) less than 1 month before a blood sample was collected, or any systemic therapy less than 1 week before a blood sample was collected. Informed consent was obtained from all subjects involved in the study.

Blood was drawn before the SRT (CTC1) and 4 to 5 weeks after the SRT (CTC2) for BCBM. The blood samples for the CTC1 and CTC2 assessments were collected on the same days as the cranial magnetic resonance imaging (MRI) for the SRT planning and the first follow-up MRI, respectively. The relationship between the number of irradiated BCBM with SRT and the CTC at both timepoints was evaluated and correlated with the impact on brain disease control during the follow-up period.

Detailed descriptions of the SRT procedure and the follow-up MRI imaging have already been published [6,12]. Briefly, all participants were treated within 1 week after the MRI and computed tomography (CT) imaging for the SRT planning by a linear accelerator with micromultileaf collimator, cone beam CT, and robotic couch. Follow-up MRI was performed 4 to 5 weeks after the SRT, then every 3 months during the first year and every 4 months in the second year, unless an earlier schedule was clinically indicated. Imaging evaluators were blinded to the CTC analysis and CTC evaluators were blinded to the imaging analysis.

### 2.2. CTC Assessment

The assessment of the CTC was performed by the ISET (Isolation by SizE of Tumors, Rarecells, Paris, France) method. Briefly, 10 mL of blood was collected in EDTA tubes, kept under homogenization, and then diluted 1:10 with a filtration buffer. After that, the blood was transferred to the ISET block and filtered through a polycarbonate membrane with 8 µm diameter cylindrical pores, which are shorter than the CTC diameter. Next, the membranes were washed with phosphate-buffered saline, decoupled off the block, and stored at −20 °C until the analysis. CTC were counted per 1 mL of blood and characterized according to 5 criteria: negativity for CD45 staining, nucleus size >12 µm, hyperchromatic and irregular nuclei, visible cytoplasm, and nuclear-to-cytoplasm ratio >80%.

The expression of HER2 was evaluated by immunocytochemistry, which was performed using the ISET membranes. Briefly, the membranes containing the captured CTC were cut and placed in 24-well plates for antigenic recovery, followed by hydration. The cells were permeabilized with Triton 0.2% in phosphate-buffered saline (PBS), and endogenous peroxidase was blocked with 3% hydrogen peroxide. The membrane spots were then subjected to double-labeled immunocytochemistry. The antibody anti-HER2 (1:50 dilution, lot G0207S; CusaBio, Houston, TX, USA) was used. To confirm that the analyzed CTC were not leukocytes, we used an anti-CD45 antibody (1:100 dilution, lot F1222Y; CusaBio). All antibodies were separately diluted in PBS and 10% fetal bovine serum. To amplify the antibody signals, the spots were incubated with the Envision G/2 Doublestain rabbit/mouse system (Agilent Technologies, Santa Clara, CA, USA), followed by incubation with DAB and Permanent Red (Agilent Technologies). The cells were then stained with hematoxylin and analyzed under an optical microscope (BX61; Olympus, Tokyo, Japan).

We recorded positive HER2 expression in the CTC, regardless of the intensity. It is important to emphasize that sometimes, even when the patient had many CTC, some ISET spots did not show CTC. Therefore, when the spot had no CTC, it was not possible to evaluate protein expression.

### 2.3. Endpoints

**Figure 1:**
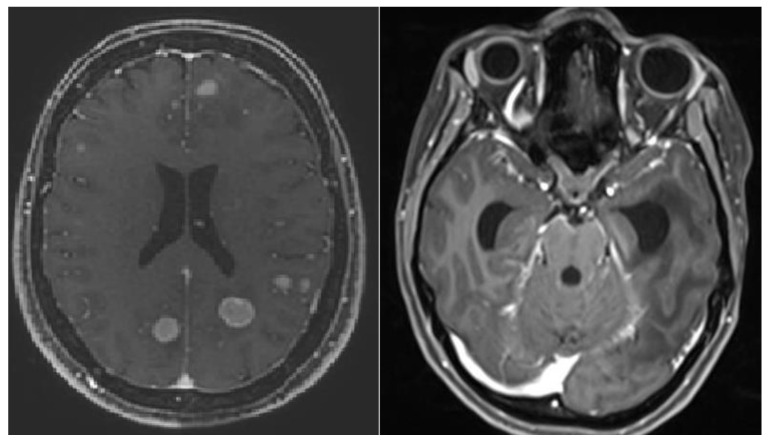
Representative MRI of diffuse distant brain failure: progression with more than 4 new lesions (**left**) and meningeal carcinomatosis (**right**).

The primary endpoint was distant brain failure-free survival (DBFFS), defined as the time from the date of the SRT to the development of any new lesion suggestive of BCBM outside the previous planning target volume, not present on prior scans or visible in a minimum of 2 projections on the follow-up MRI, according to the Response Assessment in the Neuro-Oncology Brain Metastases working group [13]. Diffuse distant brain failure-free survival (D-DBFFS) was defined as progression with more than 4 new BCBM or meningeal carcinomatosis (Figure 1). OS was defined as the time from the date of the SRT to the date of death.

### 2.4. Statistical Analysis

The statistical analysis was performed using R software version 3.5 (R Foundation for Statistical Computing, Vienna, Austria) and SPSS Statistics version 23 (IBM, Armonk, NY, USA). Baseline characteristics were expressed as descriptive statistics. DBFFS and OS were estimated by the Kaplan–Meier test and analyzed using the log-rank test. For the multivariate analysis of prognostic factors that affected DBFFS and OS, the Cox proportional model was adjusted. The significance level was fixed at 5% for all tests. The study closed in February 2018, and the data set was locked on 30 October 2018.

## 3. Results

### 3.1. Baseline Characteristics

Baseline characteristics, including patient and treatment data, are listed in Table 1. Between November 2016 and February 2018, 39 women were accrued.

Patients underwent SRT with radiosurgery (SRS) or stereotactic fractionated radiotherapy (SFRT) in 68% (27) and 31% (12) of cases, respectively, with a median prescribed dose of 20 Gy (15–22) with SRS and 27.5 Gy (25–30) in five fractions with SFRT.

### 3.2. CTC Detection and HER2 Expression

Before the SRT, CTC were detected in all 39 patients, and after SRT, in 34 out of 35 patients (97%). The median numbers of CTC1 and CTC2 were 2 CTC/mL and 2.33 CTC/mL, respectively (*p* = 0.357).

The discordance rate between the PIP and HER expression in CTC1 and CTC2 is shown in Table 2. Only 1 out of 15 evaluable patients who were PIP HER2-positive expressed HER2 in the CTC1 (6.7%), and 2 out of 14 evaluable patients who were PIP HER2-positive expressed HER2 in the CTC2 (14%). Nine patients expressed HER2 in their CTC1 or CTC2, of which two were PIP HER2-positive.

### 3.3. Survival Analysis

In the 39 evaluable patients, the median follow-up (FU) time was 16.6 months (95% CI, 14.8–19.4) for OS. In the 36 patients evaluable for DBFFS, the median FU time was 14.6 months (95% CI, 11.1–18.1). During the FU, DBF was observed in 15 patients, of which 6 had D-DBF (3 patients with more than four new BCBM and 3 patients with meningeal carcinomatosis) and 16 patients died. The median DBFFS was 15.3 (95% CI, 12.2–not reached) and the median OS was 19.5 months (95% CI, 16.1–22.9).

**Figure 2:**
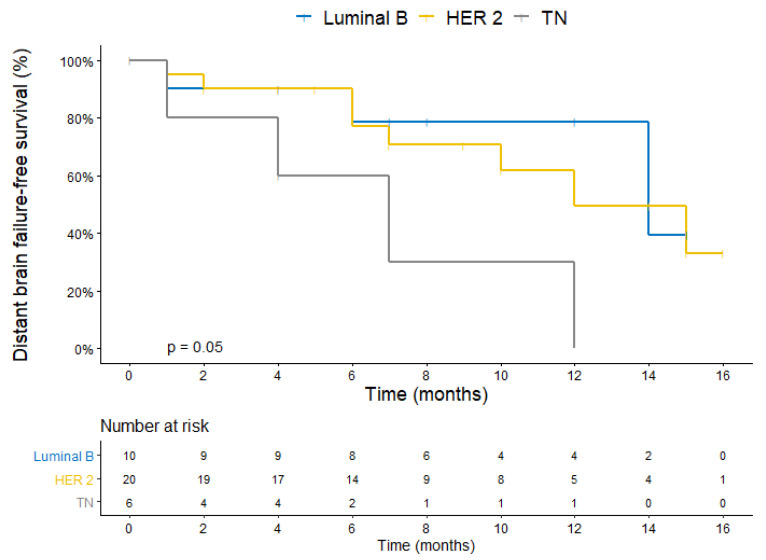
Kaplan–Meier plot for distant brain failure-free survival stratified by the primary immunophenotype.

**Figure 3:**
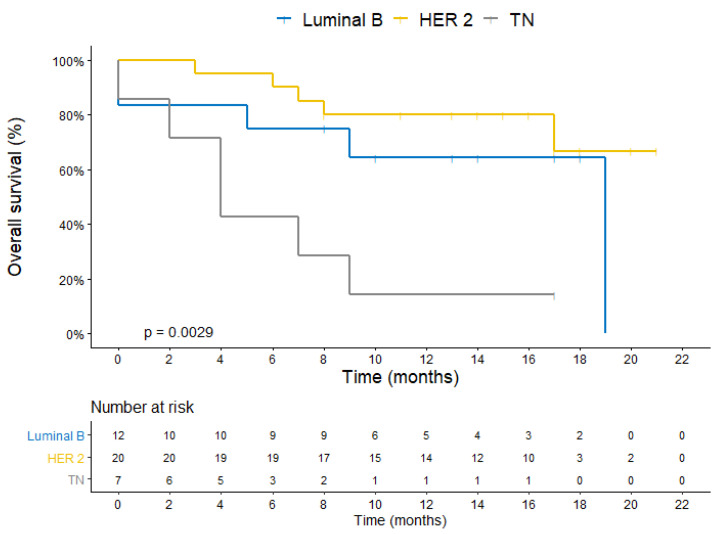
Kaplan–Meier plot for overall survival stratified by the primary immunophenotype.

The median DBFFS was 7 months in patients with a triple-negative PIP, not reaching the PIP luminal B (*p* = 0.04), and being HER2-positive (*p* = 0.03) (Figure 2). The median OS was 4.8 months in patients with a triple-negative PIP, not reaching the PIP luminal B (*p* = 0.05), and being HER2-positive (*p* = 0.002) (Figure 3).

**Figure 4:**
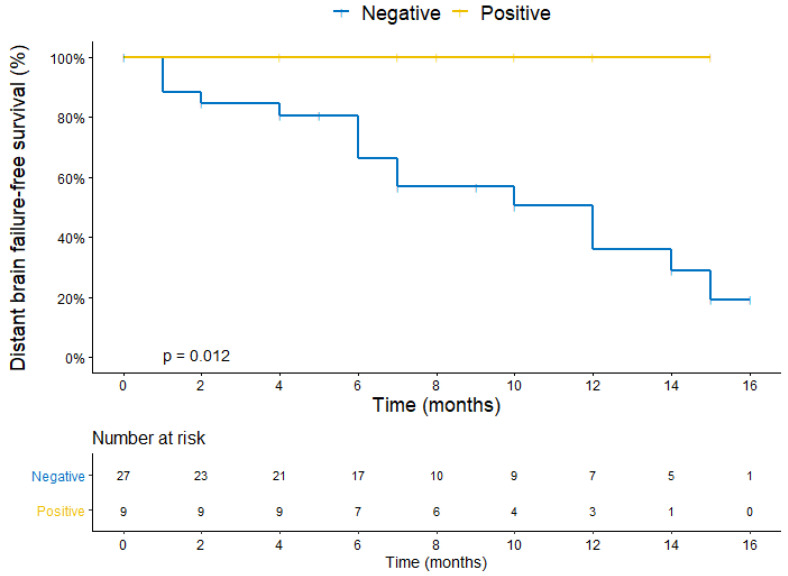
Kaplan–Meier plot for distant brain failure-free survival stratified by HER2 expression in the CTC1 or CTC2.

**Figure 5:**
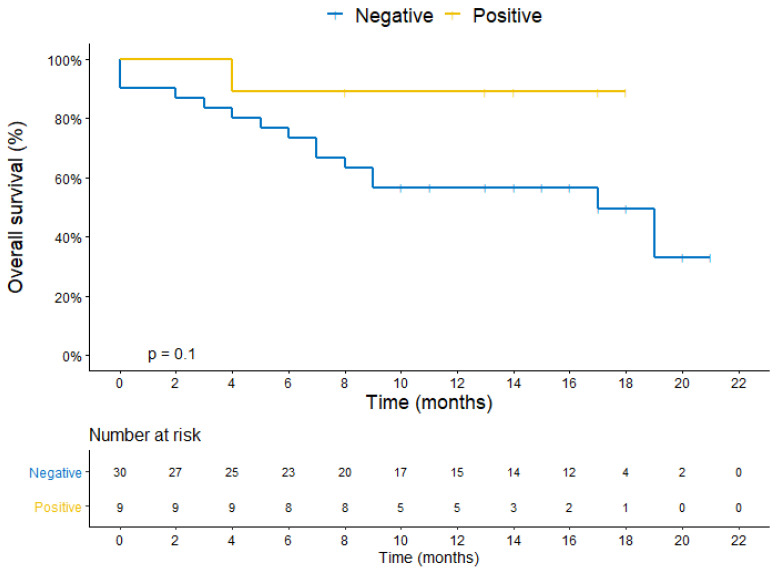
Kaplan–Meier plot for overall survival stratified by HER2 expression in the CTC1 or CTC2.

The median DBFFS was 10 months in 30 patients who were HER2-negative and not reached in 9 patients who were HER2-positive in the CTC (*p* = 0.012) (Figure 4). The median OS was 17 months in patients who wereHER2-negative and not reached in patients who were HER2-positive in the CTC (*p* = 0.104) (Figure 5).

On the multivariate analysis, the DBFFS was superior in patients who were PIP HER2-positive (HR 0.128, 95% CI 0.025–0.534; *p* = 0.013) and OS was superior in patients who werePIP HER2-positive (HR 0.073, 95% CI 0.018–0.288; *p* < 0.0001) and luminal B (HR 0.224, 95% CI 0.062–0.816; *p* = 0.023). The status of expression of HER2 in CTC was not included in the Cox model for DBFFS due to a lack of events in patients who were HER2-positive in CTC.

## 4. Discussion

This prospective study addressed an underexplored topic, that is, the heterogeneity of HER2 expression in CTC and its impact on brain disease control, with a pragmatic approach. Different to the previous initial study [6], in this exploratory analysis, we did not evaluate the number of CTC nor the additional expressions of proteins within HER2. More importantly than the number of CTC, the genetic and phenotypic characterizations have potential applications as a predictive and prognostic biomarker and a guide for clinical decisions, especially for patients with BCBM.

The significantly longer DBFFS for patients who expressed HER2 in their CTC observed in our study is in line with previous findings of lower DBF in patients being PIP HER2-positive [6,14,15]. Vern-Gross et al. evaluated the patterns of failure of BCBM after SRT and revealed that the breast cancer subtype predicted the DBF rate, with significantly lower rates for patients with PIP HER2 and luminal HER2 [14]. Mills et al. reported a similar finding in a more recent cohort, like ours [6], suggesting that the new and more available anti-HER2 therapies did not change the pattern of DBF [15].

However, the HER2 discordance between the PIP and BCBM is not so uncommon, and the same could be observed for the HER2 discordance between the PIP and CTC. In a systematic review and meta-analysis of BCBM and primary tumor receptor discordance, 12% out of 540 patients exhibited discordant HER2 status, with a HER2 gain in 9% and a HER2 loss in 3% [10]. In a retrospective analysis of 107 patients, Wallwiener et al. revealed that the overall accuracy between HER2 expression in CTC, PIP HER2 status and metastatic tumor tissue HER2 status was 69% and 74%, respectively [16].

The next question is whether changing the HER2 status would impact the prognosis. In 219 patients with BCBM, Hulsbergen et al. described a discordance prevalence of 10.4% for HER2 between the PIP and BCBM, with a gain of expression of 8% and a loss of expression of 2.4%. Interestingly, patients gaining HER2 status showed a nonsignificant tendency toward improved OS [17]. Wallwiener et al. reported a significantly longer median progression-free survival (PFS) in patients who were HER2-positive versus HER2-negative CTC (7.4 versus 4.34 months; *p* = 0.035), but no significant difference for OS (13.7 versus 8.7 months; *p* = 0.287) [16]. Similarly, Zhang et al. verified that 62.1% of patients had a heterogeneous HER2 status between the primary tumor and CTC, and that the median PFS of the CTC HER2-positive patients was significantly longer than for the CTC HER2-negative patients (8.5 versus 3.5 months; *p* < 0.001) [18].

Curiously, in patients with HER2-negative metastatic breast cancer screened for participation in the DETECT study and analyzed using the *CellSearch* system, the presence of ≥1 CTC with strong HER2 staining was associated with a shorter OS. A possible explanation for this contrasting finding is that patients receiving HER2-directed treatment based on the detection of HER2-positive CTC were not included in the survival analysis [19]. In our study with a pragmatic approach, 38% of patients were receiving HER2-targeted therapy, which might have altered the clinical course of disease and improved the outcome for patients. Wang et al. evaluated the prognostic value of the HER2 status on CTC in advanced-stage breast cancer patients with HER2-negative tumors and found that, among the patients with high-risk HER2-positive CTC, those who received anti-HER2 targeted therapies had an improved PFS compared with those who did not [20].

Regarding the HER2 discordance between the PIP and CTC, we observed that 6.7% of the evaluable patients who were PIP HER2-positive expressed HER2 in their CTC1 and 14% expressed in their CTC2, whereas 12% of the evaluable patients who were PIP HER2-negative expressed HER2 in their CTC1 and 46% expressed in their CTC2. This was similar to the rates of 32% and 49% of HER2-positive CTC in patients who were PIP HER2-negative using *CellSearch* and the *AdnaTest BreastCancer*, respectively, identified in a prospective, multicenter trial [21]. Using the same platforms, Aktas et al. revealed that primary lesions and CTC expressed a concordant HER2 in 59% of cases, and for metastases and CTC, the concordance value was 67% for HER2 with the *AdnaTest*. Using *CellSearch*, no significant concordance was observed when HER2’s effect on CTC was compared with HER2’s effect on primary lesions and metastases [22]. Jordan et al. identified dynamic and spontaneous interconversion of HER2 expression in CTC, with cells of one phenotype descending cells of the opposite, and they noticed that differential proliferation promoted a HER2-positive status, while oxidative stress or cytotoxic chemotherapy enhanced transition to the HER2-negative phenotype [11].

Since 75% (15 of 20) of patients who were PIP HER2-positive in our study received HER2-targeted therapy, most of them with dual blockade, the low proportion of CTC HER2-positive suggests CTC HER2-negative was induced by therapy. A similar trend was reported in the clinical trial GeparQuattro, in which CTC were evaluated before and after neoadjuvant therapy for patients with nonmetastatic breast cancer. In patients who were PIP HER2-positive, 27.3% expressed HER2 in their CTC before NT and 30% expressed it after NT [23]. In patients who were PIP HER2-negative, Meng et al. showed that HER2 gene amplification in CTC was acquired in 37.5% (9 of 24) of patients as their breast cancer progressed. Moreover, four out of nine patients were treated with an anti-HER2 therapy; one had a complete response and two had a partial response [24]. In a recent study by our same group with a different cohort of patients, out of 4 patients who were PIP HER2-positive, none had HER2-positive CTC, while out of 18 patients who were PIP HER2-negative, 17 were CTC HER2-negative, with an accuracy of 77.3%, mainly due the higher concordance between the PIP and CTC HER2-negative, but due less to low concordance between the PIP and CTC HER2-positive, which is probably a consequence of selection of CTC HER2-negative induced by anti-HER2 targeted therapy [25].

An additional plausible explanation for the heterogeneity of HER2 expression in CTC and between CTC and primary breast cancer is evolutionary clonality. Riebensahm et al. compared the copy number alteration (CNA) profiles of CTC to the corresponding tumor tissue in three patients and observed that the CNA profile of CTC resembled those of primary tumors. However, alterations in known pathways to brain metastases formation were identified, suggesting a clonal selection of CTC for brain metastases [26].

While it is known that HER2 expression in the primary tumor, brain metastases, or CTC are associated with a higher OS [2,6,10], the data on the impact of HER2 expression in CTC on DBF in our study are unprecedent as far as we are concerned. The significantly longer DBFFS in patients with HER2-positive CTC at any moment suggests that a focal approach with SRT for BCBM should be prioritized over WBRT to avoid the potential cognitive and quality of life impairments. In fact, there is a trend to consider SRT for patients with a better prognosis, or when active systemic therapy to the central nervous system is available [27,28,29]. The prospective observational study JLGK0901, involving 1194 patients with 1 to 10 newly diagnosed brain metastases treated exclusively with SRS, revealed that the median OS did not differ between the patients with 2 to 4 tumors and those with 5 to 19 (10.8 months in both groups), suggesting that SRT might be a suitable alternative for patients with up to 10 brain metastases [30]. In this context, a reliable biomarker to predict early DBF would be applicable in a frequent clinical scenario, which is the definition between SRT and WBRT to optimize the control of BCBM and attenuate potential cognitive impairment [31].

This proof-of-principle research has some limitations. The small sample size of our study may have impaired the reliability of the data analysis. In addition, the immunocytochemistry applied to evaluate the expressions of multiple proteins in CTC is a challenging technique, with sensitivity variability and a risk of cross-reactivity with the different antibodies. Moreover, there was inhomogeneous systemic therapy with possible different clinical impacts on the number of CTC and brain disease control, although the last day of the systemic treatment was defined only 1 week before the blood sample for the CTC analysis was collected.

Interestingly, new studies have been evaluating potential CTC-based biomarkers of brain metastases in the peripheral blood and brain environment, such as the cerebrospinal fluid. A recent hypothesis-generating study found different expression patterns of CD44 and CD74 between the brain environment and the peripheral blood, supporting the different role of these proteins in the brain and in the circulatory system [32]. We have reported a counterintuitive finding of significantly longer DBFFS in patients with a higher number of CTC in the blood, suggesting intercompartmental migration of CTC between the brain, the cerebrospinal fluid, and the blood [6]. Therefore, some CTC would be more amenable to metastasize to the brain, whereas another sort of CTC would be prone to disseminate to extracranial sites, and the HER2 status in CTC may be a biomarker of this intercompartmental behavior, which is to be confirmed in further studies. In fact, a contemporary prospective study investigated the role of a HER2-positive CTC ratio in the process of metastatic spread and identified two cohorts: HER2-positive with high CTC (the ratio defined as the sum of high fluorescence intensity HER2-positive CTC divided by the total number of HER2-positive CTC > 0.75) and HER2-positive with low CTC (ratio ≤ 0.75). Patients who were HER2-positive with low CTC were correlated to multiple sites of metastatic involvement, with a particular significant tropism toward liver, lung and lymph nodes [33].

More than the detection and enumeration of CTC, the development of different CTC capture technologies has enabled the assessment of its genomes, transcriptomes, and proteomes. Hence, single-CTC multi-omics can detail intercellular heterogeneity and provide the evolutionary profile as well as insights into specific metastatic organ-tropism [34].

## 5. Conclusions

In conclusion, our findings suggest that the status of HER2 expression in CTC has a potential role as a biomarker of DBF, refining treatment selection for patients with BCBM. It would allow a more objective approach to decide between focal and whole-brain radiotherapy or even the administration of the new antibody–drug conjugate anti-HER2 with central nervous system activity.

## Figures and Tables

**Table 1:** Baseline characteristics.

Median Age, Years (Range)	
54 (34–70)	
Immunophenotype (%)	
HER2-positive	20 (51)
Luminal B	12 (31)
Triple negative	7 (18)
Diagnosis-specific graded prognostic assessment (%)	
0–1	1 (2.5)
1.5–2	6 (15.5)
2.5–3	6 (15.5)
3.5–4	26 (66.5)
Extracranial metastases status (%)	
Absent	6 (15.5)
Progressive	17 (43.5)
Stable	16 (41)
Previous treatment to the brain (%)	
None	18 (46)
SRT	9 (23)
Surgery	5 (13)
WBRT	4 (10)
Surgery and SRT or WBRT	3 (8)
Systemic therapy before CTC1 (%)	
None	3 (8)
Hormonal therapy	9 (23)
Chemotherapy	12 (31)
HER2-targeted therapy	15 (38)

**Table 2:** Comparison of HER2 expression in the CTC1 and CTC2 and corresponding primary tumors.

		Primary Immunophenotype		Total (%)
		HER2-Negative (%)	HER2-Positive (%)	
CTC1	HER2-negative	15 (47)	14 (44)	29 (90.5)
	HER2-positive	2 (6.5)	1 (3)	3 (9.5)
	Total	17 (53)	15 (47)	32 (100)
CTC2	HER2-negative	7 (26)	12 (44.5)	19 (70.5)
	HER2-positive	6 (22)	2 (7.5)	8 (29.5)
	Total	13 (48)	14 (52)	27 (100)

## Data Availability

The data that support the findings of this study are available from the corresponding author upon reasonable request.
